# Deactivation of SARS-CoV-2 with pulsed-xenon ultraviolet light: Implications for environmental COVID-19 control

**DOI:** 10.1017/ice.2020.399

**Published:** 2020-08-03

**Authors:** Sarah E. Simmons, Ricardo Carrion, Kendra J. Alfson, Hilary M. Staples, Chetan Jinadatha, William R. Jarvis, Priya Sampathkumar, Roy F. Chemaly, Fareed Khawaja, Mark Povroznik, Stephanie Jackson, Keith S. Kaye, Robert M. Rodriguez, Mark A. Stibich

**Affiliations:** 1 Xenex Disinfection Services, San Antonio, Texas; 2 Department of Virology and Immunology, Texas Biomedical Research Institute, San Antonio, Texas; 3 Department of Medicine, Central Texas Veterans Healthcare System, Temple, Texas; 4 Department of Medicine, College of Medicine, Texas A&M Health Science Center, Bryan, Texas; 5 Jason and Jarvis Associates, Hilton Head Island, South Carolina; 6 Mayo Clinic, Rochester, Minnesota; 7 Department of Infectious Diseases, Infection Control and Employee Health, The University of Texas MD Anderson Cancer Center, Houston, Texas; 8 Department of Quality, WVU Medicine: United Hospital Center, Bridgeport, West Virginia; 9 Department of Quality, HonorHealth, Scottsdale, Arizona; 10 School of Medicine, Department of Infectious Diseases, University of Michigan, Ann Arbor, Michigan; 11 Department of Emergency Medicine, University of California San Francisco, San Francisco, California

## Abstract

**Objectives:**

Prolonged survival of severe acute respiratory syndrome coronavirus 2 (SARS-CoV-2) on environmental surfaces and personal protective equipment may lead to these surfaces transmitting this pathogen to others. We sought to determine the effectiveness of a pulsed-xenon ultraviolet (PX-UV) disinfection system in reducing the load of SARS-CoV-2 on hard surfaces and N95 respirators.

**Methods:**

Chamber slides and N95 respirator material were directly inoculated with SARS-CoV-2 and were exposed to different durations of PX-UV.

**Results:**

For hard surfaces, disinfection for 1, 2, and 5 minutes resulted in 3.53 log_10_, >4.54 log_10_, and >4.12 log_10_ reductions in viral load, respectively. For N95 respirators, disinfection for 5 minutes resulted in >4.79 log_10_ reduction in viral load. PX-UV significantly reduced SARS-CoV-2 on hard surfaces and N95 respirators.

**Conclusion:**

With the potential to rapidly disinfectant environmental surfaces and N95 respirators, PX-UV devices are a promising technology to reduce environmental and personal protective equipment bioburden and to enhance both healthcare worker and patient safety by reducing the risk of exposure to SARS-CoV-2.

Individuals with coronavirus disease 2019 (COVID-19), asymptomatic carriers of severe acute respiratory syndrome coronavirus 2 (SARS-CoV-2), and viral super-shedders readily contaminate the environment, which may lead to transmission to other patients and healthcare workers (HCWs).^1–4^ Shedding can come from both respiratory and fecal secretions.^5–7^ A recent report examining the survival of SARS-CoV-2 in the healthcare environment found that 13 of 15 (87%) room sites sampled were positive for SARS-CoV-2 by polymerase chain reaction assay.^2^ SARS-CoV-2 has been demonstrated to survive on surfaces, such as plastic and steel, for up to 3 days.^8^ This extensive spreading and prolonged survival opens the possibility of indirect transmission of SARS-CoV-2 from surfaces, which is consistent with data from prior coronavirus outbreaks such as severe acute respiratory syndrome (SARS) and Middle Eastern respiratory syndrome (MERS).^9–12^ Infection clusters of SARS-CoV-2 have been reported where no direct contact with an infected individual has occurred but several persons became infected.^13,14^

These and other data document that the environment poses a risk of SARS-CoV-2 transmission. It is difficult to ensure that manual cleaning and disinfection occur consistently in healthcare settings,^15^ and cleaning personnel could be at increased risk of exposure to SARS-CoV-2 during their performance of manual cleaning of healthcare facilities. Thus, we sought to determine the efficacy of ultraviolet C (UV-C)–enhanced environmental disinfection against SARS-CoV-2.

In addition, due to an acute shortage of N95 respirators and other personal protective equipment (PPE), healthcare facility personnel have been using a variety of methods (UV-C, hydrogen peroxide, heat, radiation) to disinfect and reuse these PPE.^16^ Given that such PPE are repeatedly used by HCWs, it is important to determine whether such disinfection is effective in reducing SARS-CoV-2 on such PPE, so that HCWs are not exposing themselves to this virus with PPE reuse.

Prevention of healthcare-associated infections was a priority before the COVID-19 pandemic, and it is even more important now to prevent SARS-CoV-2 transmission to patients and HCWs. In addition, enhanced SARS-CoV-2 transmission risks exist in other settings such as nursing homes, meat processing plants, prisons and jails, schools, restaurants, and other workplaces.

UV-C has promise as a means of environmental control for SARS-CoV-2. To understand the potential of UV-C as a tool in the pandemic, we must first understand the effect of UV-C on SARS-CoV-2 and the necessary operating time to reduce the bioburden of SARS-CoV-2 in the environment. Herein, we present the results of a laboratory study that assessed the efficacy of full-germicidal-spectrum UV-C from a pulsed-xenon source (PX-UV) on SARS-CoV-2 on hard surfaces and N95 respirators.

## Methods

### Cells and virus

Vero E6 cells (VERO C1008, cat. no. NR-596; BEI Resources, Washington, DC) were grown in Dulbecco’s modified essential media (DMEM; Gibco, Invitrogen, Grand Island, NY) with 10% heat-inactivated fetal bovine serum (FBS; Gibco) at 37°C with 5% CO_2_.

The SARS-CoV-2 working stock was generated from isolate USA-WA1/2020, obtained from BEI resources (cat. no. NR-52281; GenBank accession no.: MN985325.1). Virus was passaged once to generate a master stock using the following methods. Vero E6 cells were infected at a multiplicity of infection of ~0.001 in DMEM containing 2% FBS in T150 flasks. Viral supernatant was harvested 3 days after infection when the cells exhibited 3+ cytopathic effects, and the supernatant was clarified by low-speed centrifugation. This master stock was confirmed to be SARS-CoV-2 via deep sequencing and was stored at <−65°C in 500 ± 50 µL aliquots containing DMEM with 10% FBS. A working stock was generated by infecting Vero E6 cells at a multiplicity of infection of 0.01 in DMEM containing 2% FBS, in T225 flasks. Viral supernatant was harvested 3 days after infection, clarified by low-speed centrifugation, and further concentrated by centrifugation at 12,000×*g* for 3 hours. The supernatant was removed from the concentrate, and the remaining pelleted material was pooled to generate the stock used in these experiments. The viral titer was 1.3 × 10^7^ plaque-forming units (PFU)/mL.

### PX-UV device testing at Texas Biomedical Research Institute experimental design

The procedures and processes utilized to execute the experiment were approved by the Texas Biomedical Research Institute institutional review boards. No human participants were involved in this study. The experiments to test the antiviral effects of PX-UV robot model PXUV4D (Xenex Disinfection Services, San Antonio, TX) on SARS-CoV-2 were performed at the Texas Biomedical Research Institute. Test surfaces (ie, carriers) included a hard surface (8-well chamber slides) and a soft surface (N95 respirator, 3M Model 1860). Test surfaces were inoculated with 0.020 mL of virus, deposited in a single drop, and spread with a pipette tip. Test surfaces were then dried for 55 minutes in a laminar-flow hood under ambient conditions. Three carriers per test surface were harvested at time zero to determine the starting viral titer per carrier type, and they were stored on wet ice while PX-UV exposure occurred for the remaining carriers. There was a 30-minute difference between controls being harvested and all exposures being completed. The robot was placed 1 m from the test surfaces. Test surfaces were placed vertically to be parallel with the lamp and exposed in triplicate to the PX-UV robot for the specified contact time. Chamber slides were exposed for 1-, 2-, or 5-minute durations, and the N95 respirator carriers were exposed for 5-minute durations (Table 1). After the exposure period, virus was immediately harvested in 150 µL DMEM supplemented with 2% FBS. Recovered virus was stored on wet ice until processing. Recovered virus was serially diluted (100 µL was used to prepare a 1:1 dilution and 50 µL was used to prepare serial 10-fold dilutions). This material was subjected to plaque assay as described below. Viral titers were determined as PFU/mL in starting material harvested from the carriers. Data were analyzed by authors R.C., K.A., and H.S.

**Table 1.** Experimental Design

Test Microorganism	Test Device	Treatment Distance	Test Surface	Contact Time	Number
SARS-CoV-2	PX-UV device	1 m	Chamber slide	0 min	3
			Chamber slide	1 min	3
			Chamber slide	2 min	3
			Chamber slide	5 min	3
			N95 respirator	0 min	3
			N95 respirator	5 min	3

Note. SARS-CoV-2, severe acute respiratory syndrome coronavirus 2; PX-UV, pulsed-xenon ultraviolet light.

### Determination of viral titers

Viral titers were determined by plaque assay using a methylcellulose and crystal violet assay.^17,18^ Vero E6 cells were seeded in 12-well tissue-culture plates in DMEM with 10% heat-inactivated FBS at a density of 2 × 10^5^ cells per well. Positive control samples (ie, material from slide and N95 respirator with no UV exposure) were serially diluted 10-fold, and test samples were diluted 1:1 and serially 10-fold. Dilutions were prepared in DMEM containing 2% FBS. Media were removed from the plates and 100 µL of each dilution was added to the corresponding well in duplicate. A negative control plate was prepared as well. The plates were incubated for one hour at 37°C with 5% CO_2_, with constant rocking. After incubation, media were removed from the wells and a 2-mL primary overlay consisting of DMEM with 2% heat-inactivated FBS and 30% methylcellulose (Sigma-Aldrich, St Louis, MO) was added. Plates were then incubated at 37°C with 5% CO_2_ for 3 days. After 3 days, the overlay was removed and 10% neutral buffered formalin (Sigma-Aldrich) was added to each well to fix the cells. After fixation, formalin was removed and the plates were washed in 1X phosphate-buffered saline (Gibco). To stain the plates, ~500 µL of crystal violet stain (Ricca Chemical, Arlington, TX) was added to each well, and plates were incubated at room temperature for 10 minutes. The plates were then washed in fresh water and allowed to air dry before plaques were counted to determine a final titer.

## Results

The controls for the hard surfaces carriers averaged a titer of 6.20 PFU/mL (log_10_). In contrast, the PX-UV exposed hard surfaces had 2.67 PFU/mL (log_10_) (3.56 log_10_ reduction,99.97%) at 1 minute of PX-UV exposure, <1.66 PFU/mL (log_10_) (>4.54 log_10_ reduction, 99.997%) at 2 minutes of PX-UV exposure, and <2.08 PFU/mL (log_10_) (>4.12 log_10_ reduction, 99.992%) at 5 minutes of PX-UV exposure (Table 2). The detection threshold of the experimental methods was 1.3 PFU/mL (log_10_), and that value was inserted when the levels of SARS-CoV-2 on the carriers were undetectable.

**Table 2.** Impact of Pulsed-Xenon Ultraviolet Light on SARS-CoV-2 Inoculated Onto Hard Surfaces

Test Surface	UV-C Exposure Time, min	Viral Titer [PFU/mL (Log_10_)] per Carrier			Average	% Reduction	Log_10_ Reduction Relative to Respective Controls
		1	2	3			
Slide	0	6.04	6.28	6.28	6.20	N/A	N/A
Slide	1	3	2.45	2.56	2.67	99.97	3.53
Slide	2	2.38	<1.3	<1.3	<1.66	>99.997	>4.54
Slide	5	<1.3	2.15	2.8	<2.08	>99.992	>4.12

Note. UV-C, ultraviolet C light; PFU, plaque-forming units; N/A, not applicable.

Next, we evaluated the impact of PX-UV on SARS-CoV-2 inoculated on N95 respirators. Control titers averaged at 6.35 PFU/mL (log_10_). Inoculated N95 respirators exposed to 5 minutes of PX-UV showed <1.56 PFU/mL (log_10_), or a >4.79 log_10_ reduction (99.998%) (Table 3). The detection threshold of the experimental methods was 1.3 PFU/mL (log_10_), and that value was inserted when the levels of SARS-CoV-2 on the carriers were undetectable.

**Table 3.** Impact of Pulsed-Xenon Ultraviolet Light on SARS-CoV-2 Inoculated on N95 Respirators

Test Surface	UV-C Exposure Time, min	Viral Titer [PFU/mL (Log_10_)] per Carrier			Average	% Reduction	Log_10_ Reduction Relative to Respective Controls
		1	2	3			
N95 Respirator	0	6.84	6.04	6.18	6.35	N/A	N/A
N95 Respirator	5	<1.3	<1.3	2.08	<1.56	>99.998	>4.79

Note. UV-C, ultraviolet C light; PFU, plaque-forming units; N/A, not applicable.

## Discussion

The results from our study demonstrate that the rapid disinfection times from PX-UV devices can effectively reduce the viable load of SARS-CoV-2 in a laboratory setting on both chamber slides and N95 respirators. To our knowledge, this PX-UV device is the first no-touch disinfection system to show efficacy directly against SARS-CoV-2 on hard surfaces. UV-C has been the most common method of PPE disinfection in response to the pandemic, despite conflicting data regarding its efficacy. Our study is also the first to demonstrate that PX-UV specifically is effective in reducing SARS-CoV-2 on N95 respirators. The results of tests demonstrating that disinfection with PX-UV will not impact the fit or function of the respirators are available from the respirator manufacturer.^19^ Under the current FDA guidance for reuse of N95 respirators, the level of disinfection that PX-UV devices provide could be used for tier 3 bioburden reduction. Respirators that are disinfected using this method are suitable only for single users to supplement CDC reuse recommendations.^20^

Use of PX-UV is not a novel concept; it has been deployed for hospital-acquired infection prevention, including multidrug-resistant organisms such as methicillin-resistant *Staphylococcus aureus* and *Clostridioides difficile*.^21–24^ The device is most commonly used during the terminal cleaning of patient rooms, after manual disinfection using an EPA-registered disinfectant. With SARS-CoV-2, there will be additional target areas for disinfection. Given that emergency departments and SARS-CoV-2 testing centers are the primary sites for triage and evaluation of suspected SARS-CoV-2 patients, use of PX-UV should be considered throughout these areas, including triage, patient rooms, radiology suites, and patient bathrooms. Considering the potential for secondary transmission, non–patient-care areas (ie, lobbies, waiting rooms, staff break rooms, cafeterias, and staff on-call rooms) should be considered for disinfection as well. Portable medical equipment also should also be considered as possible vectors for transmitting SARS-CoV-2. Equipment such as mobile work stations, vital signs machines, wheel chairs, and intravenous pumps, can become heavily contaminated with routine use.^25,26^ Disinfection of portable medical equipment with a PX-UV device resulted in a 94% reduction in bacterial load.^27^ Our demonstration that brief cycles of PX-UV disinfection are effective in decreasing SARS-CoV-2 attests the feasibility of its use in these settings.

Our study has several limitations. We did not evaluate the direct effect of PX-UV on existing healthcare environmental contamination but rather high virion concentration in a laboratory setting. Our inoculum exceeded the level of SARS-CoV-2 contamination that would be seen in a routine clinical healthcare environment. It is likely that in such clinical environments, the impact of the PX-UV in reducing environmental bioburden would be even greater.

The results from our study cannot be generalized to other UV light sources because UV-C from a PX-UV system is fundamentally different from that produced by other UV disinfection systems that rely on low-pressure mercury vapor lamps or light-emitting diode sources.^28^ UV-C from a PX-UV system produces broad-spectrum wavelength light that covers the entire germicidal UV spectrum, from 200 to 280 nm,^29^ potentially creating more viricidal effect than the wavelengths produced by other narrow-spectrum sources.^30^

We found that PX-UV significantly reduces SARS-CoV-2 on hard surfaces and N95 respirators. With the potential to rapidly disinfectant environmental surfaces and N95 respirators, PX-UV devices are a promising technology for the reduction of environmental and PPE bioburden.
